# Available, Bed-sided, Comprehensive (ABC) score to a diagnosis of Methicillin-resistant *Staphylococcus aureus* infection: a derivation and validation study

**DOI:** 10.1186/s12879-017-2919-2

**Published:** 2018-01-08

**Authors:** Nori Yoshioka, Matsuo Deguchi, Hideharu Hagiya, Hisao Yoshida, Norihisa Yamamoto, Shoji Hashimoto, Yukihiro Akeda, Kazunori Tomono

**Affiliations:** Division of Infection Control and Prevention, Osaka University Hospital, 2-15 Yamadaoka, Suita, Osaka, 565-0871 Japan

**Keywords:** Clinical diagnosis, Diagnostic score, Methicillin-resistant *Staphylococcus aureus*, Nosocomial infection

## Abstract

**Background:**

Methicillin-resistant *Staphylococcus aureus* (MRSA) infections continue to be a leading problem in health care facilities worldwide.

**Methods:**

This single-center retrospective cohort study consisted of a derivation phase and a validation phase. The derivation phase included all patients admitted to Osaka University Hospital between May 2010 and April 2011. We proposed a provisional available, bed-sided, comprehensive (ABC) score, and evaluated its accuracy using the clinical diagnosis as a reference. We subsequently revised ABC scores based on *k* coefficient scores of each variable; this revision was validated by applying it to another patient population.

**Results:**

A total of 172 patients and 154 cases were enrolled in the derivation and validation studies, respectively. The revised ABC score consisted of four simple variables: type of clinical specimen (1 to 3 points), Gram-staining result (1 point), presence of local inflammation (2 points), and a systemic inflammatory response (2 points). A revised score of ≥5 points was sensitive (93.8%) and specific (90.6%), and the area under the receiver-operating curve was 0.969 (95% CI; 0.957–1).

**Conclusions:**

We developed a simple and comprehensive scoring system for diagnosis of nosocomial MRSA infections; this system is applicable in a wide variety of situations.

**Electronic supplementary material:**

The online version of this article (10.1186/s12879-017-2919-2) contains supplementary material, which is available to authorized users.

## Background

Nosocomial infections caused by antimicrobial-resistant bacteria are associated with high mortality and morbidity. Methicillin-resistant *Staphylococcus aureus* (MRSA) is a leading cause of nosocomial infections worldwide [1, 2], although recent studies have indicated that the incidence of invasive MRSA infections is declining [3, 4]. Due to its potential pathogenicity and multimodal infective forms, MRSA infections cause substantial mortality and healthcare costs [5]. For example, in the case of surgical site infections, the MRSA-caused mortality rate is over three times greater than that caused by methicillin-susceptible *S. aureus* [6]. Mortality associated with MRSA bacteremia remains as high as 30% [7]. Thus, early diagnosis followed by appropriate treatment is essential for this potentially fatal infection.

Overuse of antibiotics can lead to the emergence of antimicrobial-resistant pathogens. As for MRSA, an increasing prevalence of strains with higher MIC for vancomycin has been reported [8]. Furthermore, vancomycin [9], linezolid [10], and daptomycin [11] resistances have been reported worldwide. Thus, therapeutic options have become limited, and the importance of antibiotic stewardship is increasingly apparent.

For the appropriate use of antimicrobials, reliable diagnostic criteria that can clearly differentiate active infections from colonization are important. Clinical practice guidelines for MRSA infections are well established [12], but diagnosis remains difficult in real practice. Though previous studies proposed various scoring models to stratify the risk of contracting MRSA among patients with pulmonary or bloodstream infections, these scoring models are limited in predicting the presence of MRSA infection rather than differentiating active MRSA infection from colonization status [13, 14]. MRSA colonizes multiple body sites and causes various infections in humans [15]. It is therefore imperative to develop a comprehensive scoring system targeting multi-organ infections.

From the perspective of infection control activity, nosocomial transmission of MRSA should be carefully monitored in hospitals. Infection control practitioners are not necessarily medical doctors in Japan; this category of professionals includes nurses, laboratory technicians, pharmacologists, and even clerks. During surveillance, distinguishing between active infection and colonization is essential, but is an arduous task for these professionals. A user-friendly method of MRSA surveillance is thus required. The present study aimed to establish an available, bed-sided, comprehensive (ABC) score to differentiate various types of active MRSA infections from its colonization, by stratifying patient data with simple criteria.

## Methods

### Study design and setting

We conducted this retrospective observational study at Osaka University Hospital, a tertiary medical center (1086 beds) in Japan. The study consisted of a derivation phase and a validation phase. The derivation phase enrolled all patients consecutively admitted to the hospital, from May 2010 to April 2011, with any positive culture for MRSA during hospitalization. We first proposed a provisional version of the ABC score to differentiate active infection from colonization. We subsequently evaluated the provisional ABC score, and developed a revised version. In the validation phase, the revised ABC score was applied to a distinct patient cohort in the hospital. The study protocol was accepted by the Institutional Review Board of Osaka University Hospital (No. 15576). Informed consent was waived because we retrospectively collected data without using any individually identifying information or applying any intervention.

### Definition of clinical diagnosis for MRSA infections

Patients with MRSA strains were identified from records of the clinical microbiology laboratory, and electronic health records of all of the cases were carefully reviewed by an infectious disease (ID) specialist. A clinical diagnosis of active MRSA infection was comprehensively determined by referring to the following criteria: (i) inflammation and clinical signs were apparent at the site where MRSA was detected, (ii) systemic inflammation was apparent (fever, elevation of peripheral white blood cells [WBC] or serum C-reactive protein [CRP]), and (iii) inflammation was alleviated upon treatment with MRSA-targeted antimicrobials.

### Bacterial identification

Bacterial identification and antimicrobial susceptibility testing were performed using a MicroScan Walkaway Plus System (Beckman Coulter, Brea, CA, USA).

### Establishing and evaluating the provisional ABC score

A provisional version of the ABC score was proposed according to current guidelines and our clinical experience (Additional file 1). In this version, 3 points were assigned when MRSA was detected in aseptic samples such as blood, ascites, pleural fluid, pancreatic fluid, spinal fluid, abscesses, or wounds. No additional points were assigned for cases in which MRSA was isolated from other samples. Two points were assigned when the amount of MRSA was judged microscopically to be ≥2+ on a Gram-staining smear or > than 10^5^ CFU/mL in bacterial culture. In addition, 2 points were assigned when a Gram-staining smear showed neutrophil aggregation or phagocytosis of the bacteria. One point was respectively given for the presence of indicators of inflammation (redness, hotness, swelling, or tenderness), a systemic inflammatory response (high fever, chills, rigors, hypotension, decreased urine output, or elevated peripheral WBC [> 9400/μL] or serum CRP [> 0.2 mg/dL]). Cases with 3 points or less were determined to be colonization, whereas those with 4 points or more were classified as active infection. We evaluated the accuracy of these provisional scores by comparing them with clinical diagnoses.

### Revising the provisional ABC score

To efficiently improve the scoring system, the validity of the additional points given to the provisional scores was investigated. For each clinical sample (nasal, pharyngeal, expectorated sputum, aspirated sputum, blood, drained pus, surgical site pus, non-surgical site pus, and others), sensitivity and specificity of the provisional score to clinical diagnosis were calculated. The samples were stratified into four groups: Group 0, ratios of colonization and active infection more than 50% and less than 50%, respectively; Group 1, ratios of both colonization and active infection less than 50%; Group 2, ratios of colonization and active infection less than 50% and more than 50%, respectively; and Group 3, ratios of colonization and active infection less than 50% and over 70%, respectively. Results of the bacterial counts in culture were divided into four categories (−/+/2+/3+). Gram-staining results were subdivided in terms of WBC, red blood cells, gram-positive cocci, gram-positive bacilli, gram-negative cocci, gram-negative bacilli (−/+/2+/3+), superiority of gram-positive cocci to other bacteria, and phagocytosis of gram-positive cocci (presence or absence). Evidence of local and systemic inflammation and the elevation of inflammatory markers (WBC and CRP) were categorized as either presence or absence.

All of the cases were classified as colonization, undetermined, or active infection by a single rater according to the clinical diagnosis. The sensitivity, specificity, diagnostic concordance rate, *k* coefficient, and positive and negative likelihood ratios were calculated for the applicable variables in the provisional score. In cases where sensitivity, specificity, and the diagnostic concordance rate were all above 50%, additional points were assigned, which were weighted according to the *k* coefficient score. In a previous report [16], the value of the *k* statistic was interpreted as follows: < 0.01, poor; 0 to 0.20, slight; 0.21 to 0.40, fair; 0.41 to 0.60, moderate; 0.61 to 0.80, substantial; and 0.81 to 1, almost perfect. Based on this interpretation, we gave 1 point for a *k* coefficient ranging from 0.01 to 0.40, 2 points for a *k* coefficient ranging from 0.41 to 0.80, and 3 points for a *k* coefficient ranging from 0.81 to 1.00.

### Validation study

To validate the accuracy of the revised scoring system, we conducted a retrospective validation study using another patient population of the hospital. Clinical data from patients who became positive for MRSA between April and August of 2015 were evaluated by two independent staffs of the hospital’s Division of Infection Control and Prevention. The revised ABC score was also applied to the patient data by a distinct researcher.

### Data analysis

The diagnostic concordance rate was defined as a matching rate between the ABC score and the clinical diagnosis. Undetermined cases were excluded from the calculation in the derivation phase. The *k* coefficient was calculated and evaluated as previously described by Landis et al. [16]. Receiver operating characteristic (ROC) curves were generated and the area under the curve (AUC) was used to determine the cutoffs for the scoring system. The statistical analyses were performed using the EZR software, which is a modified version of R Commander (version 2.2–5) based on R (version 3.3.1) [17]. We applied the Chi-squared test for nominal data, and the Mann–Whitney U test for continuous variables. A *p* value <0.05 was considered statistically significant.

## Results

During the study’s derivation phase, a total of 172 cases were identified as MRSA carriers and were included in the analysis. Of these cases, 106 (61.6%) were male, and the median [interquartile range (IQR)] age was 63.5 years [46.75, 76]. The patient background as well as admission wards is summarized in Additional file 2. MRSA was isolated from blood (13), nasal (29), pharyngeal (18), expectorated sputum (15), aspirated sputum (38), pus/exudate (12), urine (6), drainage-associated (6), ascites (3), stool (3), intravascular catheter (2), and other (11) samples. The “other” category included pleural effusion, synovial fluid, periosteum, cornea, and skin, aural, and vaginal discharge samples.

### Evaluation of the provisional ABC scores

Based on the clinical diagnosis, there were 48 active infections and 98 colonization cases. Twenty-six cases were judged to be undetermined, and excluded from the evaluation of the provisional score. Of the 48 cases of active infection, the provisional score classified 1 case (2.1%) as colonization, and 47 cases (97.9%) as active infections. On the other hand, of the 98 cases of colonization, the provisional score classified 60 cases (61.2%) as colonization, and 38 cases (38.8%) as active infections. Accordingly, the sensitivity, specificity, diagnostic concordance rate, positive predictive value, and negative predictive value of the provisional ABC score were 97.9%, 61.2%, 73.3%, 55.3%, and 98.4%, respectively.

### Evaluating the provisional score and developing the revised ABC score

All of the clinical specimens were classified into three categories (colonization, undetermined cases, or active infection) according to the clinical diagnoses (Additional file 3). Subsequently, the types of clinical specimen were divided into four groups (Groups 0 to 3) based on the ratio of active infections to colonization (Table 1). The sensitivity, specificity, diagnostic concordance rate, *k* coefficient, positive likelihood ratio, and negative likelihood ratio for each categorization are shown in Additional file 4.

**Table 1:** Categorized clinical specimens into 4 groups on the basis of clinical diagnosis

Clinical diagnosis N (%)	Group 0				Group 1			Group 2	Group 3	
	Nasal 29	Pharyngeal 18	Expectorated sputum 15	Other non-aseptic specimen 17	Surgical site 14	Pus 12	Aspirated Sputum 36	Drained 7	Blood 14	Other aseptic specimen 10
Colonization (%)	28 (96.6)	18 (100)	10 (66.7)	14 (82.4)	5 (35.7)	4 (33.3)	17 (47.2)	0 (0.0)	2 (14.3)	0 (0.0)
Undetermined (%)	1 (3.4)	0 (0.0)	2 (13.3)	2 (11.8)	7 (50.0)	4 (33.3)	6 (16.7)	3 (42.9)	0 (0.0)	1 (10.0)
Active Infection (%)	0 (0.0)	0 (0.0)	3 (20.0)	1 (5.9)	2 (14.3)	4 (33.3)	13 (36.1)	4 (57.1)	12 (85.7)	9 (90.0)

The revised ABC score was developed according to these results (Table 2). In the revised version, additional points for clinical specimens were applied as follows: 3 points for blood and other aseptic specimens; 2 points for drained pus; and 1 point for aspirated sputum and surgical site pus as MRSA sources. For the findings of Gram-staining smearing, 1 point was given when the white blood cell counts or gram-positive cocci were more than (2+), and when the number of gram-positive cocci was greater than that of other bacteria. In addition, 2 more points were assigned based on signs of local or systemic inflammation. No additional points were given to bacterial counts in culture, other findings based on Gram-staining, or serum inflammatory markers such as WBC and CRP.

**Table 2:** The revised ABC score

	Additional points	
	Yes	No
1. Clinical specimen		
Blood or other aseptic specimen^a^	3	0
Drained pus	2	0
Aspirated sputum or surgical site pus	1	0
2. Gram-staining		
White blood cells; ≥ 2+	1	0
Gram-positive cocci; ≥ 2+	1	0
The number of gram-positive cocci is greater than that of other bacteria	1	0
3. Local inflammation	2	0
4. Systemic inflammatory response^b^	2	0
Diagnostic criteria (Possible total points: 10)		
≤ 4 points: Colonization		
≥ 5 points: Active infection		

^a^Ascites, pleural fluid, pancreatic fluid, spinal fluid, abscess, or wound

^b^ Fever, chills, rigors, hypotension, or decreased urine output

**Fig. 1:**
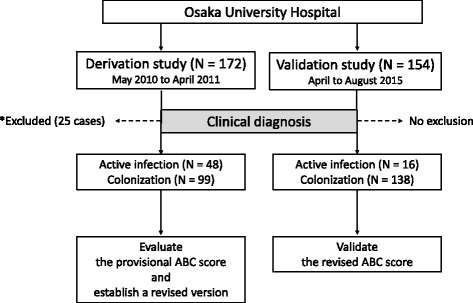
Over view of the study. Patient background of the two cohorts is demonstrated in Additional file 2

**Fig. 2:**
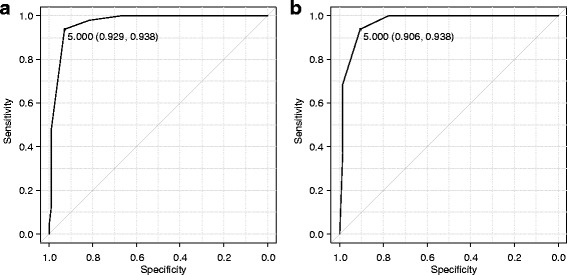
Receiver operating characteristic curves for the revised ABC score. **a** Derivation phase: sensitivity, 93.8%; specificity, 92.9%; area under the curve (AUC), 0.967 (95% CI: 0.941–0.992). **b** Validation phase: sensitivity, 93.8%; specificity, 90.6%; area under the curve (AUC), 0.979 (95% CI: 0.957–1)

We determined the cut-off for the revised ABC score to be 5 points, by obtaining an ROC curve with an AUC of 0.967 (95% CI; 0.941–0.992) (Figs. 1 and 2a). Using clinical diagnosis as the benchmark, sensitivity, specificity, diagnostic concordance rate, positive predictive values, and negative predictive values of the revised scoring system were, respectively, 93.8%, 92.9%, 93.2%, 86.5%, and 96.8%, in the derivation study. In addition, we divided the cases into two groups for subgroup analysis—a non-sterile (Group 0–2) and a sterile (Group 3) sample group. AUCs of the two groups were satisfactory: 0.973 (95% CI; 0.946–1.0) and 0.914 (95% CI; 0.829–0.999), respectively. Details of the subgroup analysis are summarized in Additional file 5 (A, B).

### Validation study

In the validation phase, 154 cases were enrolled. Of these cases, 93 (60.4%) were male, and the median age [IQR] was 64.0 years [39.25–74]. There was no significant difference between derivation and validation cohorts in terms of age distribution and sex proportion. Along with admitted department of the patients, Additional file 2 shows a comparison of the two populations.

In total, 16 active infections and 138 contamination cases were included in the analysis. A score ≥ 5 showed excellent discriminatory power, with an AUC of 0.969 (95% CI; 0.957–1) (Fig. 2b), and associated sensitivity, specificity, diagnostic concordance rate, and positive and negative predictive values of 93.8%, 90.6%, 90.9%, 53.6%, and 99.2%, respectively. Compared with the provisional version, accuracy of the revised score was improved (Table 3). The subgroup analysis for non-sterile (Group 0–2) samples in validation phase demonstrated a consistently high utility of the scoring system (AUC, 0.978; 95% CI, 0.955–1.0; sensitivity, 92.9%; specificity, 93.3%). For the sterile samples (Group 3) in the validation phase, subgroup analysis was not performed due to the small case number (*n* = 6) (Additional file 5C).

**Table 3:** Comparison of the provisional and revised scores

	*Derivation study (N = 172)*				*Validation study (N = 154)*	
Clinical diagnosis	*Provisional ABC score*		*Revised ABC score*		*Revised ABC score*	
	AI	Co	AI	Co	AI	Co
Active Infection	47	1	45	3	15	1
Colonization	38	60	7	91	13	125
Sensitivity	97.9% (47/48)		93.8% (45/48)		93.8% (15/16)	
Specificity	61.2% (60/98)		92.9% (91/98)		90.6% (125/138)	
Concordance rate	73.3% (107/146)		93.2% (136/146)		90.9% (140/154)	
Positive predictive value	55.3% (47/85)		86.5% (45/52)		53.6% (15/28)	
Negative predictive value	98.4% (60/61)		96.8% (91/94)		99.2% (125/126)	

*AI* active infection, *Co* colonization

## Discussion

We developed the ABC score as a simple approach to differentiating MRSA infections from colonization. We focused on MRSA infections because the pathogen is problematic in clinical situations worldwide. The ABC score consists of routine clinical and laboratory data that are widely available in most medical facilities. In addition, the scoring system targets all types of clinical samples; thus, it can be applied to a wide variety of clinical situations.

The ABC score can be used in many medical facilities. It consists of (i) a type of clinical sample, (ii) a Gram-staining result, (iii) a physical finding of local inflammation, and (iv) a routine laboratory result. Our scoring approach does not require any specific knowledge or experience. Risk stratification for MRSA colonization has been investigated in previous studies [18–20], because early recognition of MRSA colonization is important [21]. A previous report showed that invasive MRSA infections are frequently preceded by colonization [22]. However, there has been no reliable methodology to differentiate an active MRSA infection from MRSA colonization.

Our inclusive scoring approach is novel in that it targets every clinical sample, without being limited to infections of specific organs. MRSA is isolated from various clinical samples, and it causes systemic infections. We therefore intended to establish a systemic and comprehensive scoring approach. The results of subgroup analysis showed that our ABC score is applicable to both non-sterile and sterile samples. Delays in appropriate diagnosis can lead to poor patient prognosis, while overutilization of antibiotics can induce drug-resistance in pathogens. Appropriate antibiotic use combined with infection control can play an important role in reducing the prevalence of MRSA infections [23]. Thus, an accurate scoring system was required, and the ABC score achieved good sensitivity and specificity in the validation cohort (respectively, 93.8% and 90.6%). Due to a lack of prior studies, we cannot compare the efficacy or utility of the presently proposed system to those of other scoring methods. Further validation studies will be indispensable. However, considering the accessibility of the ABC score, it can be widely applied by medical practitioners, including non-medical doctors, who are in charge of surveillance activities in hospitals.

This kind of scoring approach may lead to future developments of diagnostic systems for various infectious diseases by non-medical doctors, or even by artificial intelligence. Several limitations of our study should be mentioned. The study was retrospective, and the number of subjects was small. The provisional scoring system was developed based on previous guidelines and our clinical experience, and other factors were not assessed by a statistical approach. In the derivation phase, only a single rater evaluated the clinical diagnosis of MRSA infection. Our approach for recruiting and weighting new predictors (based on sensitivity, specificity, diagnostic concordance rate, and the *kappa* statistic) may be statistically insufficient and can introduce a risk of model over-fitting. Microbiological process differences (Gram staining and bacterial culture) among laboratories or even technologists may affect the results of the scoring. Clinically, a possibility of active infection should not be negated easily based only on the result of the ABC score. Especially, isolation of MRSA from sterile sites, such as blood, should be carefully evaluated, even if the result of scoring was indicative of colonization. Inappropriate interpretation of the scoring might delay a proper treatment for MRSA active infection. In addition, 15.1% (26/172) of the cases in the derivation study were excluded from the analysis; this exclusion might have misled the development of an accurate scoring system. Although validation of the revised score should have been performed in a prospective manner, time constraints allowed for no alternative but retrospective validation. A prospective multi-centered study is thus required to confirm the universal utility of our scoring method.

## Conclusions

In conclusion, we developed an available, bed-sided, comprehensive (ABC) score to diagnose MRSA infections. Differentiating active infections from colonization is important for better patient management, antimicrobial stewardship, and infection control activities in hospitals. The ABC score consists of simple factors and covers systemic infections; professionals at medical facilities can easily and widely apply this system.

## Additional files

Additional file 1: The provisional ABC score. (PDF 52 kb)

Additional file 2: Comparison of the two patient cohorts. (PDF 149 kb)

Additional file 3: Clinical diagnosis associated with each specimen in the derivation cohort. (PDF 48 kb)

Additional file 4: Evaluation of each subjects with scores applied. (PDF 100 kb)

Additional file 5: Receiver operating characteristic curves for the provisional score of the derivation phase. (PDF 399 kb)

## Abbreviations

ABC: Available, bed-sided, comprehensive
AUC: Area under the curve
CRP: C-reactive protein
ID: Infectious disease
MRSA: Methicillin-resistant *Staphylococcus aureus*
ROC: Receiver operating characteristic
WBC: White blood cells

## References

[1] Hidron AI, Edwards JR, Patel J, Horan TC, Sievert DM, Pollock DA, Fridkin SK, National Healthcare Safety Network Team; Participating National Healthcare Safety Network Facilities (2008). NHSN annual update: antimicrobial-resistant pathogens associated with healthcare-associated infections: annual summary of data reported to the National Healthcare Safety Network at the Centers for Disease Control and Prevention, 2006-2007. Infect Control Hosp Epidemiol.

[2] Stefani S, Chung DR, Lindsay JA, Friedrich AW, Kearns AM, Westh H, Mackenzie FM (2012). Meticillin-resistant *Staphylococcus aureus* (MRSA): global epidemiology and harmonisation of typing methods. Int J Antimicrob Agents.

[3] Burton DC, Edwards JR, Horan TC, Jernigan JA, Fridkin SK (2009). Methicillin-resistant *Staphylococcus aureus* central line-associated bloodstream infections in US intensive care units, 1997-2007. JAMA.

[4] Kallen AJ, Mu Y, Bulens S, Reingold A, Petit S, Gershman K, Ray SM, Harrison LH, Lynfield R, Dumyati G, Townes JM, Schaffner W, Patel PR, Fridkin SK (2010). Active Bacterial Core surveillance (ABCs) MRSA investigators of the emerging infections program. Health care-associated invasive MRSA infections, 2005-2008. JAMA.

[5] Klevens RM, Morrison MA, Nadle J, Petit S, Gershman K, Ray S, Harrison LH, Lynfield R, Dumyati G, Townes JM, Craig AS, Zell ER, Fosheim GE, McDougal LK, Carey RB (2007). Fridkin SK; active bacterial Core surveillance (ABCs) MRSA investigators. Invasive methicillin-resistant *Staphylococcus aureus* infections in the United States. JAMA.

[6] Engemann JJ, Carmeli Y, Cosgrove SE, Fowler VG, Bronstein MZ, Trivette SL, Briggs JP, Sexton DJ, Kaye KS (2003). Adverse clinical and economic outcomes attributable to methicillin resistance among patients with *Staphylococcus aureus* surgical site infection. Clin Infect Dis.

[7] Gasch O, Ayats J, Angeles Dominguez M, Tubau F, Liñares J, Peña C, Grau I, Pallarés R, Gudiol F, Ariza J, Pujol M (2011). Epidemiology of methicillin-resistant *Staphylococcus aureus* (MRSA) bloodstream infection: secular trends over 19 years at a university hospital. Medicine (Baltimore).

[8] Sader HS, Fey PD, Limaye AP, Madinger N, Pankey G, Rahal J, Rybak MJ, Snydman DR, Steed LL, Waites K, Jones RN (2009). Evaluation of vancomycin and daptomycin potency trends (MIC creep) against methicillin-resistant *Staphylococcus aureus* isolates collected in nine U.S. medical centers from 2002 to 2006. Antimicrob Agents Chemother.

[9] Sievert DM, Rudrik JT, Patel JB, McDonald LC, Wilkins MJ, Hageman JC (2008). Vancomycin-resistant *Staphylococcus aureus* in the United States, 2002-2006. Clin Infect Dis.

[10] Gu B, Kelesidis T, Tsiodras S, Hindler J, Humphries RM (2013). The emerging problem of linezolid-resistant *Staphylococcus*. J Antimicrob Chemother.

[11] Stefani S, Campanile F, Santagati M, Mezzatesta ML, Cafiso V, Pacini G (2015). Insights and clinical perspectives of daptomycin resistance in *Staphylococcus aureus*: a review of the available evidence. Int J Antimicrob Agents.

[12] Liu C, Bayer A, Cosgrove SE, Daum RS, Fridkin SK, Gorwitz RJ, Kaplan SL, Karchmer AW, Levine DP, Murray BE, Rybak MJ, Talan DA, Chambers HF (2011). Clinical practice guidelines by the infectious diseases society of america for the treatment of methicillin-resistant *Staphylococcus aureus* infections in adults and children: executive summary. Clin Infect Dis.

[13] Teshome BF, Lee GC, Reveles KR, Attridge RT, Koeller J, Wang CP, Mortensen EM, Frei CR (2015). Application of a methicillin-resistant *Staphylococcus aureus* risk score for community-onset pneumonia patients and outcomes with initial treatment. BMC Infect Dis.

[14] Chen SY, Chiang WC, Ma MH, Hsueh PR, Chang SC, Fang CC, Chen SC, Chen WJ, Chie WC, Lai MS (2012). Predicting methicillin resistance among community-onset *Staphylococcus aureus* bacteremia patients with prior healthcare-associated exposure. Eur J Clin Microbiol Infect Dis.

[15] Wertheim HF, Melles DC, Vos MC, van Leeuwen W, van Belkum A, Verbrugh HA, Nouwen JL (2005). The role of nasal carriage in *Staphylococcus aureus* infections. Lancet Infect Dis.

[16] Landis JR, Koch GG (1977). The measurement of observer agreement for categorical data. Biometrics.

[17] Kanda Y (2013). Investigation of the freely available easy-to-use software 'EZR' for medical statistics. Bone Marrow Transplant.

[18] Morgan DJ, Day HR, Furuno JP, Young A, Johnson JK, Bradham DD, Perencevich EN (2010). Improving efficiency in active surveillance for methicillin-resistant *Staphylococcus aureus* or vancomycin-resistant Enterococcus at hospital admission. Infect Control Hosp Epidemiol.

[19] Harbarth S, Sax H, Fankhauser-Rodriguez C, Schrenzel J, Agostinho A, Pittet D (2006). Evaluating the probability of previously unknown carriage of MRSA at hospital admission. Am J Med.

[20] Reilly JS, Stewart S, Christie P, Allardice G, Smith A, Masterton R, Gould IM, Williams C (2010). Universal screening for meticillin-resistant *Staphylococcus aureus*: interim results from the NHS Scotland pathfinder project. J Hosp Infect.

[21] Robinson JO, Phillips M, Christiansen KJ, Pearson JC, Coombs GW, Murray RJ (2014). Knowing prior methicillin-resistant *Staphylococcus aureus* (MRSA) infection or colonization status increases the empirical use of glycopeptides in MRSA bacteraemia and may decrease mortality. Clin Microbiol Infect.

[22] Boyce JM (2001). MRSA patients: proven methods to treat colonization and infection. J Hosp Infect.

[23] Lawes T, Lopez-Lozano JM, Nebot CA, Macartney G, Subbarao-Sharma R, Dare CR, Wares KD, Gould IM (2015). Effects of national antibiotic stewardship and infection control strategies on hospital-associated and community-associated meticillin-resistant *Staphylococcus aureus* infections across a region of Scotland: a non-linear time-series study. Lancet Infect Dis.

