# Supplementary material for: Available, Bed-sided, Comprehensive (ABC) score to a diagnosis of Methicillin-resistant Staphylococcus aureus infection: a derivation and validation study
Source: BMC Infect Dis. 2018 Jan 8;18:19. doi: 10.1186/s12879-017-2919-2 (PMC5759200; doi:10.1186/s12879-017-2919-2)
Supplement: Supplementary file 1 — The provisional ABC score. (PDF 52 kb) [file 12879_2017_2919_MOESM1_ESM.pdf]

Additional file 1. The provisional ABC score.

	Points	
	Yes	No
1. MRSA isolated from blood or other aseptic samples*	3	0
2. MRSA ≧2+ by Gram-staining or ≧10^5 cfu/mL in culture	2	0
3. Neutrophil aggregation or phagocytosis shown by Gram-staining	2	0
4. Local inflammation	1	0
5. Systemic inflammatory response**	1	0
6. Elevated inflammatory markers***	1	0

Diagnostic criteria (total points possible: 10)

≦ 3 points: Colonization

≧ 4 points: Active infection

*Ascites, pleural fluid, pancreatic fluid, spinal fluid, abscess, or wound.

**Fever, chills, rigors, hypotension or decreased urine output.

**White blood cells or C-reactive protein.
