# Supplementary material for: Available, Bed-sided, Comprehensive (ABC) score to a diagnosis of Methicillin-resistant Staphylococcus aureus infection: a derivation and validation study
Source: BMC Infect Dis. 2018 Jan 8;18:19. doi: 10.1186/s12879-017-2919-2 (PMC5759200; doi:10.1186/s12879-017-2919-2)
Supplement: Supplementary file 2 — Comparison of the two patient cohorts. (PDF 149 kb) [file 12879_2017_2919_MOESM2_ESM.pdf]

Additional file 2. Comparison of the two patient cohorts.

	Derivation (N=172)	Validation (N=154)	P value
age, median [interquartile range]	63.5 [46.75, 76]	64 [39.25, 74]	0.32
sex (M/F)	106/66	93/61	0.82
Department, % (No)			
Emergency Unit	21.5 (37)	16.3 (28)	
Gastrointestinal surgery	18.0 (31)	8.1 (14)	
Cardiac surgery	14.5 (25)	16.3 (28)	
Respiratory medicine	7.0 (12)	2.3 (4)	
Pediatrics	6.4 (11)	8.1(14)	
Otolaryngology	4.7(8)	5.2 (9)	
Dermatology	4.1 (7)	5.2 (9)	
Hematology	3.5 (6)	2.3 (4)	
Respiratory surgery	2.9 (5)	2.3 (4)	
Cardiac medicine	2.9 (5)	4.1 (7)	
Geriatric and General Medicine	2.9 (5)	2.9 (5)	
Obstetrics and gynecology	2.9 (5)	2.9 (5)	
Renal medicine	1.7 (3)	0	
Urology	1.7 (3)	1.7 (3)	
Clinical Immunology	1.2 (2)	0	
Ophthalmology	0.6 (1)	1.2 (2)	
Gastrointestinal medicine	0.6 (1)	0.6 (1)	
Neurology	0.6 (1)	1.2 (2)	
Orthopedics	0.6 (1)	1.7 (3)	
Breast and Endocrine surgery	0	2.3 (4)	
Plastic surgery	0	1.7 (3)	
Pediatric surgery	0	1.7 (3)	
Neurosurgery	0	1.2 (2)	

Chi-squared test for nominal data and Mann–Whitney U test for continuous variables.
