# Supplementary material for: Available, Bed-sided, Comprehensive (ABC) score to a diagnosis of Methicillin-resistant Staphylococcus aureus infection: a derivation and validation study
Source: BMC Infect Dis. 2018 Jan 8;18:19. doi: 10.1186/s12879-017-2919-2 (PMC5759200; doi:10.1186/s12879-017-2919-2)
Supplement: Supplementary file 3 — Clinical diagnosis associated with each specimen in the derivation cohort. (PDF 48 kb) [file 12879_2017_2919_MOESM3_ESM.pdf]

Additional file 3. Clinical diagnosis associated with each specimen in the derivation cohort.

(A) Clinical specimens assigned 0 points in the provisional version

Clinical diagnosis N (%)	Total 115	Nasal 29 (25.2%)	Pharyngeal 18 (15.7%)	Expectorated sputum 15 (13.0%)	Aspirated sputum 36 (31.3%)	Others 17 (14.8%)
Colonization	76.5% (88/115)	96.6% (28/29)	100% (18/18)	66.7% (10/15)	47.2% (17/36)	82.4% (14/17)
Undetermined	8.7% (10/115)	3.4% (1/29)	0% (0/18)	13.3% (2/15)	16.7% (6/36)	11.8% (2/17)
Active Infection	14.8% (17/115)	0% (0/29)	0% (0/18)	20.0% (3/15)	36.1% (13/36)	5.9% (1/17)

(B) Clinical specimens assigned 3 points in the provisional version

Clinical diagnosis N (%)	Total 57	Blood 14 (24.6%)	Drained pus 7 (12.3%)	Surgical site 14 (24.6%)	Pus 12 (21.1%)	Others 10 (17.5%)
Colonization	19.3% (11/57)	14.3% (2/14)	0% (0/7)	35.7% (5/14)	33.3% (4/12)	0% (0/10)
Undetermined	26.3% (15/57)	0% (0/14)	42.9% (3/7)	50.0% (7/14)	33.3% (4/12)	10.0% (1/10)
Active Infection	54.4% (31/57)	85.7% (12/14)	57.1% (4/7)	14.3% (2/14)	33.3% (4/12)	90.0% (9/10)

Others include pleural effusion, synovial fluid, periosteum, cornea, skin, and aural and vaginal discharge samples.
