# Supplementary material for: Available, Bed-sided, Comprehensive (ABC) score to a diagnosis of Methicillin-resistant Staphylococcus aureus infection: a derivation and validation study
Source: BMC Infect Dis. 2018 Jan 8;18:19. doi: 10.1186/s12879-017-2919-2 (PMC5759200; doi:10.1186/s12879-017-2919-2)
Supplement: Supplementary file 4 — Evaluation of each subjects with scores applied. (PDF 100 kb) [file 12879_2017_2919_MOESM4_ESM.pdf]

Additional file 4. Evaluation of each subjects with scores applied.

	Sensitivity	Specificity	Concordance rate	κ	PLR	NLR
Categorization of clinical specimen						
Group 1	82.6% (19/23)	72.9% (70/96)	61%	0.041	3.05	0.24
Group 2	50% (4/8)	100% (70/70)	50.7%	0.64	C. I.	0.5
Group 3	84.0% (21/25)	97.2% (70/72)	62.3%	0.83	30	0.16
Bacterial count						
(≧1+)	93.8% (45/48)	14.1% (14/98)	40.4%	0.06	1.09	0.44
(≧2+)	45.8% (22/48)	58.2% (41/99)	54.1%	0.04	1.1	0.93
(≧3+)	27.1% (13/48)	79.6% (20/99)	62.3%	0.07	1.33	0.92
White blood cells						
(≧1+)	96.7% (29/30)	23.5% (8/34)	57.8%	0.19	1.26	0.14
(≧2+)	73.3% (22/30)	58.8% (20/34)	65.6%	0.32	1.78	0.45
(≧3+)	40.0% (12/30)	79.4% (27/34)	60.9%	0.20	1.94	0.76
Red blood cells						
(≧1+)	23.3% (7/30)	91.2% (31/34)	59.4%	0.15	2.65	0.84
(≧2+)	13.3% (4/30)	97.1% (33/34)	57.8%	0.11	4.59	0.89
(≧3+)	3.3% (1/30)	100% (34/34)	54.7%	0.04	C.I.	0.97
Gram-positive cocci						
(≧1+)	96.7% (29/30)	8.8% (3/34)	50%	0.05	1.06	0.38
(≧2+)	53.3% (16/30)	58.8% (20/34)	56.3%	0.12	1.29	0.79
(≧3+)	23.3% (7/30)	82.4% (28/34)	54.7%	0.06	1.32	0.93
Gram-positive bacilli						
(≧1+)	23.3% (7/30)	55.9% (19/34)	41.5%	-0.21	0.53	1.37
(≧2+)	6.7% (2/30)	82.4% (28/34)	46.9%	-0.11	0.38	1.13
(≧3+)	3.3% (1/30)	97.1% (33/34)	53.1%	<0.01	1.14	1
Gram-negative cocci						
(≧1+)	6.7% (2/30)	79.4% (27/34)	45.3%	-0.15	0.33	1.18
(≧2+)	3.3% (1/30)	97.1% (33/34)	53.1%	<0.01	1.14	1
(≧3+)	0% (0/30)	100% (34/34)	53.1%	0.00	C.I.	1
Gram-negative bacilli						
(≧1+)	36.7% (11/30)	50.0% (17/34)	43.8%	-0.13	0.73	1.27
(≧2+)	23.3% (7/30)	85.3% (29/34)	56.3%	0.09	1.59	0.9
(≧3+)	10% (3/30)	91.2% (31/34)	53.1%	0.01	1.14	0.99
Superiority of GPC						
(GPC < others)	96.7% (9/30)	8.6% (3/34)	50%	0.05	1.06	0.38
(GPC ≦ others)	83.3% (25/30)	20.6% (7/34)	50%	0.04	1.05	0.81
(GPC > others)	63.3% (19/30)	55.9% (19/34)	59.4%	0.19	1.44	0.66
Phagocytosis of GPC	33.3 %(10/30)	94.3% (32/34)	65.6%	0.28	5.84	0.71
Local inflammatory changes	72.9% (35/48)	76.0% (73/98)	74%	0.45	3.04	0.36

**Systemic reaction**	80% (36/45)	77.1% (74/96)	78%	0.53	3.49	0.26
**Inflammatory markers**	100% (47/47)	9.8% (9/89)	40.6%	0.07	1.11	0

C.I., Calculation incapacity; GPC, Gram-positive cocci; NLR, Negative likelihood ratio; PLR, Positive likelihood ratio.
