# Supplementary material for: Available, Bed-sided, Comprehensive (ABC) score to a diagnosis of Methicillin-resistant Staphylococcus aureus infection: a derivation and validation study
Source: BMC Infect Dis. 2018 Jan 8;18:19. doi: 10.1186/s12879-017-2919-2 (PMC5759200; doi:10.1186/s12879-017-2919-2)
Supplement: Supplementary file 5 — Receiver operating characteristic curves for the provisional score of the derivation phase. (PDF 399 kb) [file 12879_2017_2919_MOESM5_ESM.pdf]

Additional file 5. Receiver operating characteristic curves for the provisional score of the derivation phase.

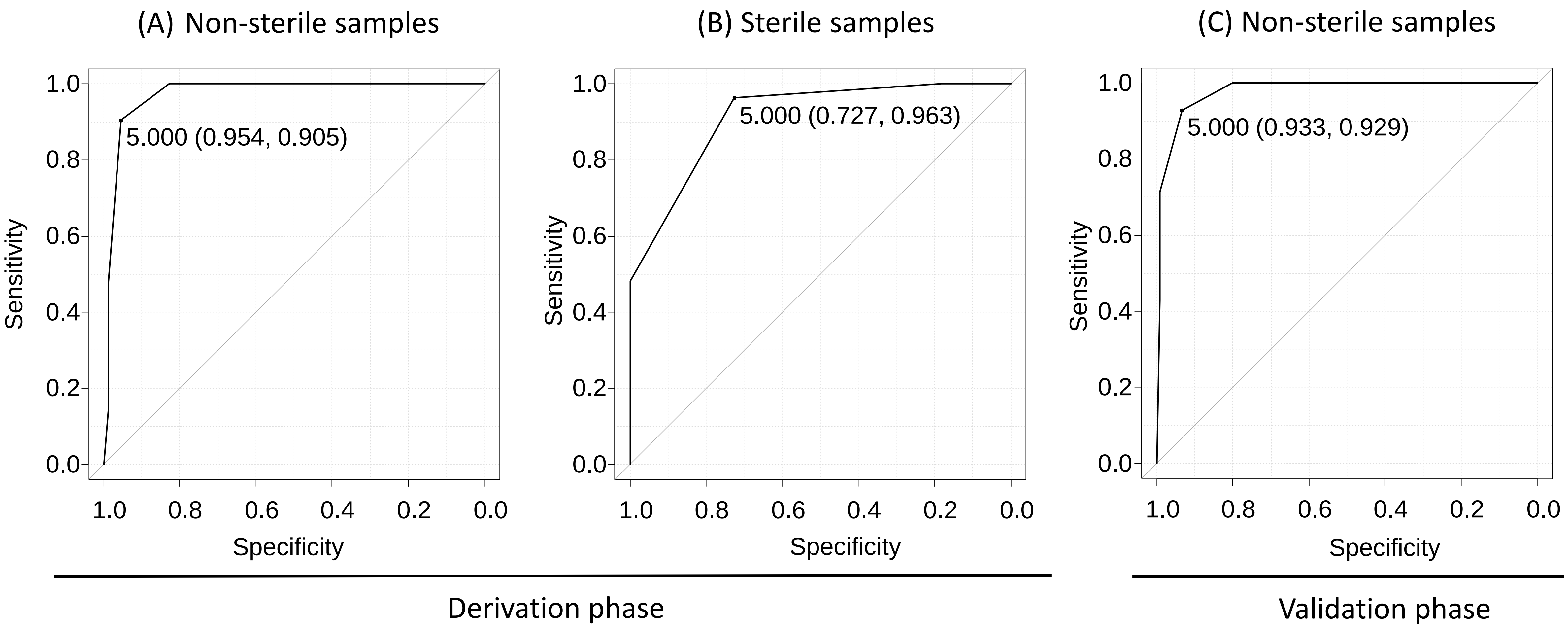

(A) sensitivity, 100%; specificity, 95.7%; PPV, 0.826; NPV, 1.0; AUC, 0.973 (95% CI: 0.946–1.0)
(B) sensitivity, 96.3%; specificity, 72.7%; PPV, 0.897; NPV, 0.889; AUC, 0.914 (95% CI: 0.829–0.999)
(C) sensitivity, 92.9%; specificity, 93.3%; PPV, 0.619; NPV, 0.991; AUC, 0.978 (95% CI: 0.955–1.0)
For the sterile samples in the validation phase, subgroup analysis was inapplicable due to the small number of the cases (n = 6).
AUC, area under the curve; PPN, positive predictive values; NPV negative predictive values; NA, not applicable.
